# Brainwave activities reflecting depressed mood: a pilot study

**DOI:** 10.1038/s41598-023-40582-y

**Published:** 2023-09-04

**Authors:** Masahiko Morita, Ryusei Otsu, Masahiro Kawasaki

**Affiliations:** 1 Graduate School of Systems and Information Engineering, University of Tsukuba, Tsukuba, Japan; 2 Present Address: Institute of Systems and Information Engineering, University of Tsukuba, Tsukuba, Ibaraki 305-8573 Japan

**Keywords:** Cognitive neuroscience, Diseases of the nervous system, Biomarkers

## Abstract

Early diagnosis and treatment of depression are desirable but currently difficult due to a lack of established biomarkers. Although biomarkers for depression based on electroencephalogram (EEG) data have long been explored, most existing methods are thought to capture cognitive decline caused by depression and are unsuccessful in detecting signs of depression. Here we report that some brainwave activities involving phase resetting reflect the depressed mood at the time, which can be easily monitored by measuring the resting EEG with eyes closed for 1 min with a few electrodes. We instructed 10 participants (nine healthy and one diagnosed with depression, aged 18–34) to record their EEG for 14–26 days. We found that indicators of depressed mood were correlated with the occurrence frequency of EEG phase resetting. For most participants, the correlation coefficients swung systematically between large positive and large negative values with respect to EEG frequency; however, the frequencies at which they were maximum or minimum differed among participants. Although this study is in the pilot phase and needs further experimentation, the results are expected to lead to innovative biomarkers for early detection of depression and may contribute to a better understanding and treatment of depression.

## Introduction

Depression is a common but serious mental disorder that requires early diagnosis and treatment. Currently, its diagnosis is primarily based on interviews and observations by specialists, and objective biomarkers for depression have not yet been established. Although easy-to-use biomarkers based on non-invasive electroencephalogram (EEG) data have been explored for more than 20 years^1,2^ and recent machine-learning-based methods can classify patients with depression and healthy controls with high accuracy^2–4^, they have not been successful in detecting signs of depression. This is possibly because their discrimination is based on brain activities that reflect long-term cognitive decline caused by depression.

For early diagnosis of depression, capturing daily changes in depressed mood is required. In addition, easy monitoring of depressed mood would be useful for assessing treatment response and follow-up of patients with depression. However, no method has been developed for such purposes.

To tackle these issues, we reanalysed data from a previous study that analysed EEG of healthy participants with high and low indicators of depressive tendencies^5^ and identified several features that could correlate with depressive tendencies. We then analysed daily EEG data from one of the authors and found that these features, especially the occurrence frequency of phase resetting^6,7^ described below, were strongly correlated with the degree of depressed mood that day.

This study aimed to show that some EEG activities are reflective of a depressed mood and to use this finding to develop practical biomarkers for early depression detection. As a first step, we conducted a pilot study to investigate the generality of the above preliminary results, mainly in healthy participants.

## Methods

### Participants

Ten volunteers (seven males and three females) aged 18–34 years (mean 23.1) recruited at the university or via a website participated in this study. As we were only making comparisons within individuals and not between individuals, and to examine how well the results were consistent across participants, we did not select participants based on age, sex, health status, or medical history. The only inclusion criterion was to complete at least 14 measurements.

Informed consent was obtained from all participants before participation. This study was conducted according to the Declaration of Helsinki and was approved by the Research Ethics Committee of the Institute of Systems and Information Engineering, University of Tsukuba (approval number 2020R443-1).

### Procedures

One measurement consisted of answering the Profile of Mood States 2nd edition (POMS-2)^8,9^ and recording EEG. The POMS-2 is a self-report questionnaire that assesses seven states: Anger–Hostility, Confusion–Bewilderment, Depression–Dejection, Fatigue–Inertia, Tension–Anxiety, Vigour–Activity, and Friendliness. In the analysis below, we used only Depression–Dejection (DD) scores because for the other scores, we did not find a clear relationship with EEG, except when they were correlated with DD. Answers to the POMS-2 questionnaire and any special notes regarding the measurement environment (e.g., loud noise), health status (e.g., fever), and medications were entered into a Google Form from a notebook PC.

EEG was performed using a 14-channel headset-type EEG device (EPOC X, Emotiv, San Francisco) with a sampling rate of 128 Hz and recorded on the PC connected to the device. The electrode positions were AF3, F3, F7, FC5, T7, P7, O1, AF4, F4, F8, FC6, T8, P8, and O2 of the 10–10 system. The EEG recordings were conducted in the resting state, with eyes open (1 min) and eyes closed (1 min). The open-eye EEG data were not used in this study because it contained more noise, and its relationship with depressed mood was less clear.

First, the measurement procedures were explained to the participants at our laboratory; subsequently, the participants performed a practice measurement under our direction, the data from which were not included in the analysis. They then took the EEG device and PC to their homes and performed self-measurements for 2–4 weeks (their convenience determined the period). There was an interval of up to 5 months between the practice measurement and the main measurements at home because of the limited number of EEG devices.

The participants were instructed to take measurements at the same time every day as much as possible. They also received instructions on how to wear the EEG device to hold the electrode position as constant as possible. However, we were unable to know the extent to which these instructions were followed, except for the time of measurement.

After all measurements were completed, the EEG device and PC were returned, and the EEG data were retrieved from the PC. The POMS data were downloaded, and seven scores (Anger–Hostility, Confusion–Bewilderment, Depression–Dejection, Fatigue–Inertia, Fatigue–Inertia, Tension–Anxiety, Vigour–Activity, and Friendliness) were calculated according to the manual^9^.

### Data processing and analysis

We collected a total of 207 measurement data, of which 11 were incomplete (five due to insufficient time of fewer than 55 s, four because of not answering the questionnaire, and two because of both the aforementioned reasons) and were therefore excluded (feature calculation is possible from shorter data, but the reliability of the phase resetting rate described below is reduced, especially at low frequencies). The remaining 196 were processed and analysed.

The first part of the data analysis is the existing time–frequency analysis using wavelet transform^10,11^. Specifically, each EEG signal, which is approximately 60 s of EEG data from one channel, was bandpass filtered between 1 and 40 Hz and then wavelet transformed with Morlet (Gabor) wavelets using functions ‘bandpass’ and ‘cwt’ of the Signal Processing Toolbox (version 8.6) and Wavelet Toolbox (version 5.6) in MATLAB (version 9.10, MathWorks, Inc.), respectively. Default values were used for the parameters of these functions, except for the sampling rate (128 Hz), passband frequency range (1–40 Hz), and type of mother wavelet for wavelet transform (Morlet).

From this wavelet-transformed signal, time series of the phase *φ*_*f*_ (*t*) (rad) and amplitude *A*_*f*_ (*t*), where *t* denotes time, were extracted for each of 40 frequencies *f* between 2 and 32 Hz (10 per octave) using functions ‘angle’ and ‘abs’ of MATLAB. In this process, the first and last 1.5 s were excluded because the first and last sections of a signal can be distorted by the wavelet transform owing to discontinuities at both ends; therefore, approximately 57-s time series was analysed.

Next, we obtained the relative angular speed *RAS*_*f*_ (*t*), which indicates how much faster or slower the phase advances than a sine wave with frequency *f*, by calculating (*φ*_*f*_ (*t*) − *φ*_*f*_ (*t* − *Δt*))/(2π*fΔt*) − 1, where *Δt* denotes the sampling interval (1/128 s). However, when *φ*_*f*_ (*t*) − *φ*_*f*_ (*t* − *Δt*) − 2π*fΔt* < − π owing to the restriction |*φ*_*f*_ (*t*)| ≤ π, *RAS*_*f*_ (*t*) was given by (*φ*_*f*_ (*t*) + 2π − *φ*_*f*_ (*t* − *Δt*))/(2π*fΔt*) − 1. When *φ*_*f*_ (*t*) − *φ*_*f*_ (*t* − *Δt*) − 2π*fΔt* > π, which rarely occurred, *RAS*_*f*_ (*t*) was given by (*φ*_*f*_ (*t*) − 2π − *φ*_*f*_ (*t* − *Δt*))/(2π*fΔt*) − 1. We also obtained the normalised amplitude *NA*_*f*_ (*t*) by *A*_*f *_(*t*) divided by its average over *t* and *f*. Then, we averaged *RAS*_*f*_ (*t*) and *NA*_*f*_ (*t*) over *t* to obtain the mean relative angular speed (MRAS) and the mean normalised amplitude (MNA), respectively.

**Figure 1:**
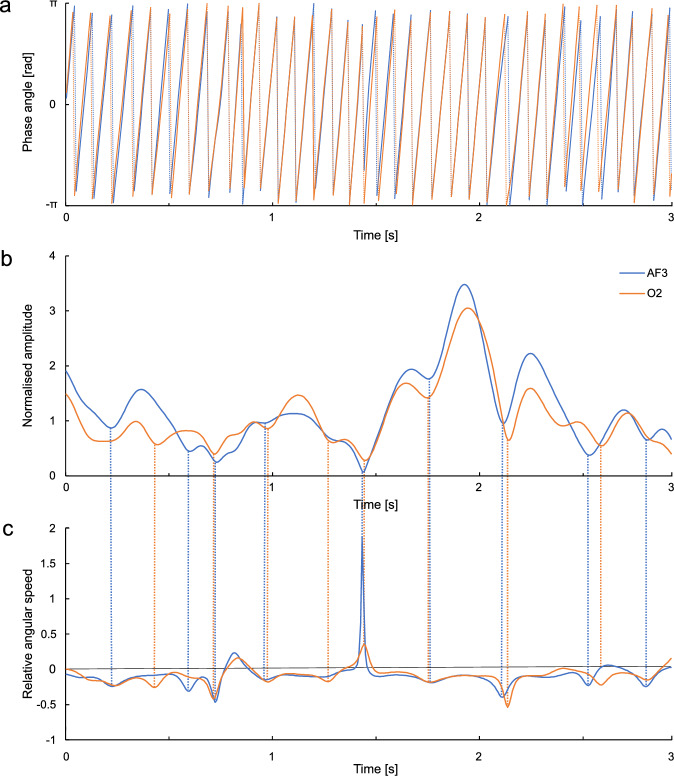
Wavelet-transformed electroencephalogram (EEG) signals at a specific frequency. The EEG signal was converted by wavelet transform to phase and amplitude at each frequency for each channel. The time courses of the phase angle (**a**) and normalised amplitude (**b**) of EEG at 12.6 Hz obtained from electrode positions AF3 (left prefrontal) and O2 (right occipital) are shown as an example. Relative angular speed (**c**) was calculated by subtracting 2π*f* from the derivative of the phase angle, where *f* denotes the frequency. In most cases, when the amplitude changes from decreasing to increasing, relative angular speed has increased or decreased rapidly, which indicates that phase resetting has occurred.

An example of *φ*_*f*_ (*t*), *NA*_*f *_(*t*), and *RAS*_*f*_ (*t*) is shown in Fig. 1. As is seen, the relative angular speed sometimes increases or decreases rapidly with a large positive or negative peak; simultaneously or slightly later, the amplitude changes from decreasing to increasing. Such changes are considered to be caused by phase resetting because they are usually observed simultaneously in multiple channels where the phases are then synchronised.

Phase resetting is generally known to occur when an external stimulus, such as sound, is presented^12^. It is thought to reflect the transmission of stimulus information^13^. However, phase resetting is also observed in the resting state with eyes closed, with little external stimulation^14^.

We counted the number of occurrences of phase resetting and calculated the frequency per cycle, which we call the phase resetting rate (PRR), and this is our original analysis. Specifically, we considered phase resetting to have occurred when *A*_*f*_ (*t*) < *A*_*f*_ (*t* − *Δt*) and *A*_*f*_ (*t*) < *A*_*f*_ (*t* + *Δt*) (i.e., the amplitude was at a minimum) and |*RAS*_*f*_ (*t*) – *MRAS*_*f*_ | > 0.05. Although not all phase resetting could be detected correctly using this criterion, the judgement was generally consistent with our visual judgement. Changing the criteria slightly altered the PRR values but did not significantly affect the main results.

We then calculated the correlation coefficients (CCs) of MRAS, MNA, and PRR with DD scores for each EEG frequency in each channel.

### Statistical analysis

As described below, the CC for PRR often swung more than once between large positive and negative values for changes in EEG frequency. To evaluate the statistical significance of this swing, a permutation test with 10,000 replicates was performed for each participant.

The test statistic was the maximum of the swing magnitude in all 14 channels, defined by the maximum difference between a positive (or negative) peak value and the average of the two adjacent negative (positive) peaks for all three consecutive peaks. The *n* DD scores were randomly shuffled and used as a permutation to calculate the test statistic if the correlation coefficient with the original scores was between − 0.5 and 0.5. The *p*-value was obtained as the proportion of permutations that yielded a greater swing magnitude than the original data.

## Results

**Figure 2:**
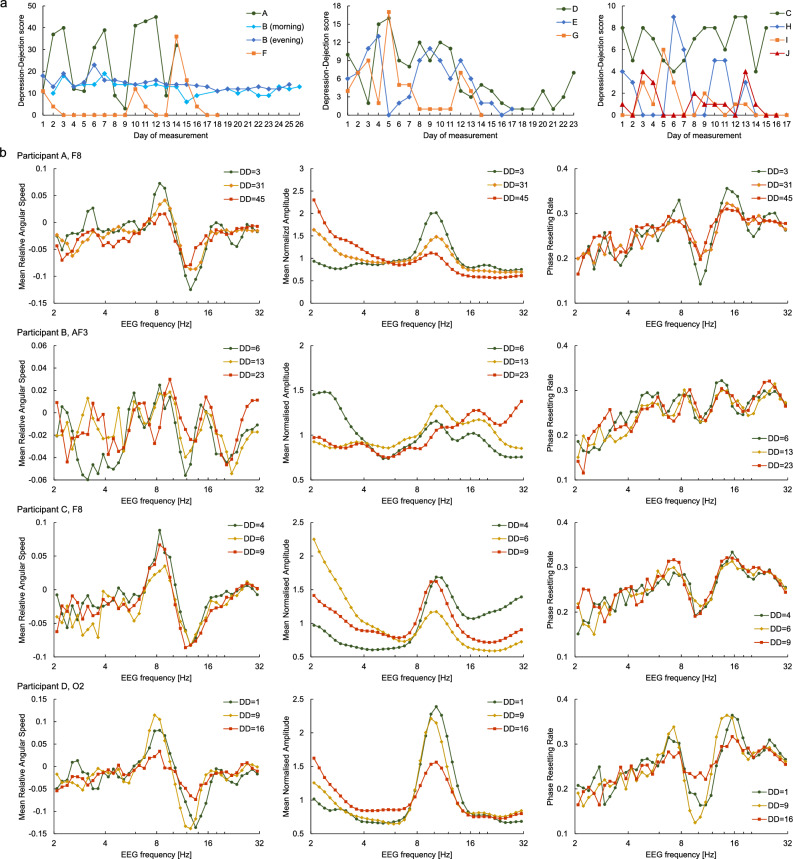
Obtained data. (**a**) Daily changes in depressed mood. Participants A–I are labelled in descending order of the mean Depression–Dejection (DD) score. Participant J reported taking antidepressants on all days, but no other participants reported taking psychotropic drugs. The days of measurement are consecutive, but in some cases, there was an interval of one to several days. Participant B voluntarily performed measurements twice daily (morning and evening) on most days. (**b**) Mean relative angular speed, mean normalised amplitude, and phase resetting rate versus electroencephalogram (EEG) frequency on a logarithmic scale. The three curves in each graph are for when DD score was the highest, lowest, and in between. See Supplementary Fig. S1 for Participants E–J.

The daily changes in the DD score for each participant are shown in Fig. 2a. Higher scores indicated a more depressed mood, and the minimum and maximum possible scores were 0 and 50, respectively. Participant A's DD score periodically increased or decreased in a cycle of approximately 5 days and exceeded 30 points on more than half of the days, indicating strong depressive tendencies. However, this participant was not depressed because the high scores lasted only for a few days. Participant J reported taking antidepressants on all days, indicating that this participant was a patient with depression. Although his/her depressed mood was considered to be suppressed by medication, we did not exclude his/her data because our analysis was performed on an individual basis. The other participants were healthy, and all showed score fluctuations of at least 5 points.

Examples of MRAS, MNA, and PRR obtained from Participants A–D are plotted against the EEG frequency *f* in Fig. 2b. MRAS had a positive peak at approximately 8 Hz and a negative peak at approximately 13 Hz, and MNA maximised near the middle of them. The PRR averaged approximately 0.25, with a negative peak at approximately 10 Hz and a positive peak at approximately 15 Hz and was negatively correlated with the mean amplitude. These properties and values were largely common to all channels and participants (Supplementary Fig. S1).

**Figure 3:**
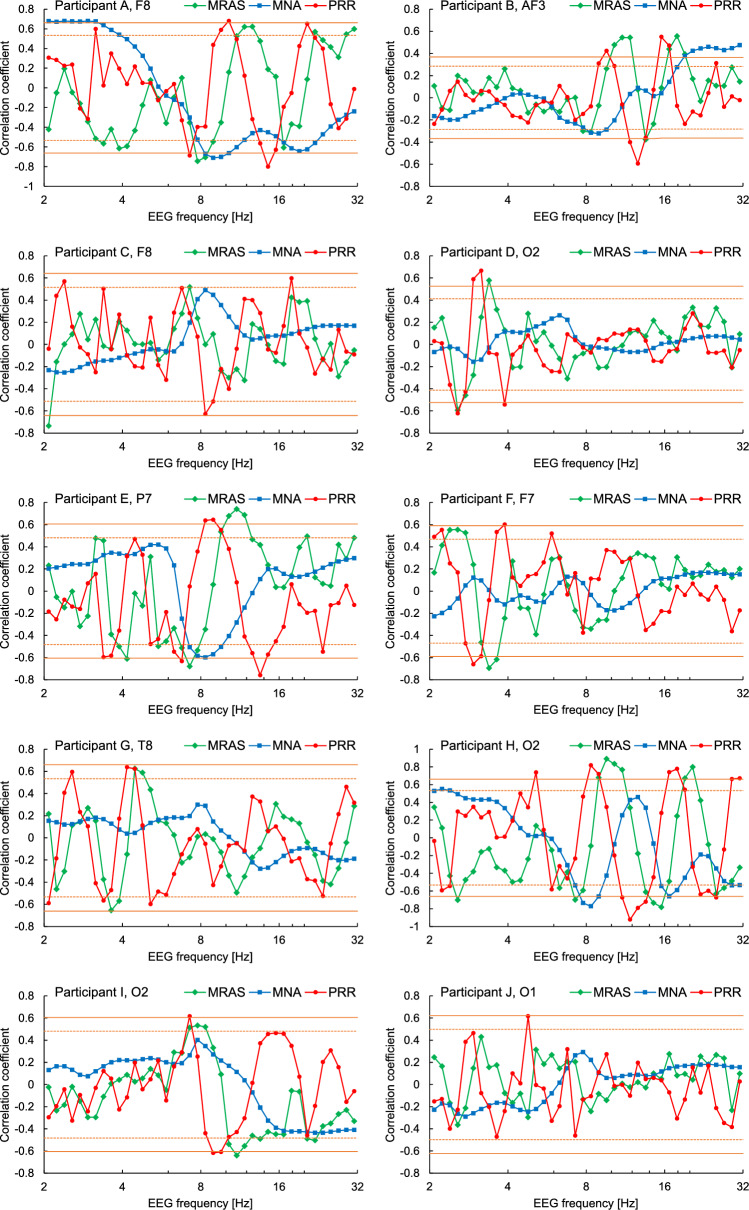
Correlation coefficients (CCs) of mean relative angular speed (MRAS), mean normalised amplitude (MNA), and phase resetting rate (PRR) with Depression–Dejection (DD) score. The values in the channel with the largest swing magnitude (see Methods for the definition) of the CC for PRR are plotted against electroencephalogram (EEG) frequency on a logarithmic scale. The orange solid and dotted lines indicate that the CC is statistically significant with *p*-values of 0.01 and 0.05, respectively, under the assumption that the samples are independent and follow a normal distribution, which is for reference only because the assumption does not necessarily hold for the DD score.

The above three features showed systematic changes in CCs with an increase in *f* (Fig. 3). For example, in channel (electrode position) F8 of Participant A, the CC for PRR increased from − 0.69 at 7.3 Hz to 0.68 at 10.3 Hz, then decreased to − 0.80 at 14.5 Hz, and again increased to 0.65 at 20.5 Hz. The curve of CC for MRAS was similar but shifted to the right. The curve for MNA had fewer peaks, and most positive and negative peaks were located near the negative and positive peaks of PRR, respectively. Overall, PRR showed the strongest correlation with DD scores, followed by MRAS and then MNA.

The positive and negative peaks of the CC curve for the PRR were roughly equally spaced on the logarithmically scaled frequency axis; that is, they often formed a geometric progression. We refer to the frequencies at which CC takes a large positive or negative peak value as positive or negative characteristic frequencies, respectively. This phenomenon was observed in many channels for most participants (Supplementary Figs. S2–S3), although the characteristic frequencies were considerably different among participants.

Table 1 shows the result of the permutation tests. The swing magnitude of the CC for PRR shown in Fig. 3 was significant (*p* < 0.05) for Participants A, B, D, E, and H, but not for Participants C, F, G, I, and J, whose DD scores did not show large variations. With the exception of participant D, all significant swings were observed around 14 Hz.

**Table 1:** Results of permutation testing for the swing of the correlation coefficient (CC) between the phase resetting rate and the Depression–Dejection scores.

Participant	Sample size	Maximum swing magnitude of CC	Result for permutations (mean ± standard deviation)	*p*-value
A	*n* = 14	1.466	1.166 ± 0.134	**0.022**
B	*n* = 48	1.080	0.682 ± 0.089	**0.0001**
C	*n* = 15	1.075	1.142 ± 0.118	0.70
D	*n* = 23	1.248	0.947 ± 0.116	**0.013**
E	*n* = 17	1.339	1.105 ± 0.112	**0.026**
F	*n* = 18	1.236	1.123 ± 0.206	0.27
G	*n* = 14	0.975	1.155 ± 0.164	0.86
H	*n* = 14	1.601	1.208 ± 0.127	**0.0016**
I	*n* = 17	1.158	1.118 ± 0.154	0.38
J	*n* = 16	0.900	1.130 ± 0.146	0.96

Significant values are in bold.

**Figure 4:**
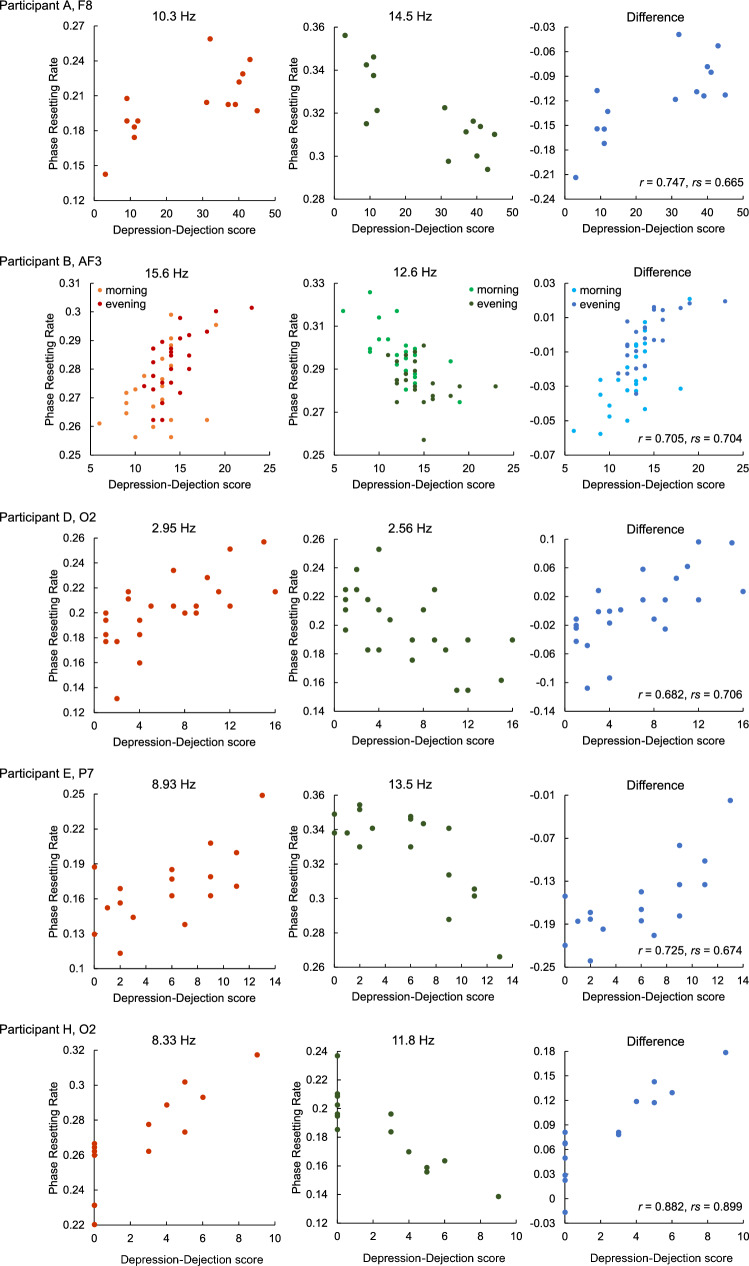
Scatter plots of phase resetting rates versus Depression–Dejection score. The left and middle panels show positive and negative characteristic frequencies, respectively, and the right panels show their difference. *r* denotes Pearson's correlation coefficient, and *rs* Spearman's rank correlation coefficient.

Figure 4 shows the PRRs at positive and negative characteristic frequencies, and their differences, in the channels where a significant swing was observed, plotted against the DD scores. This indicates that combinations of multiple PRRs obtained from different frequencies can yield a CC large enough to estimate depressed mood from EEG.

## Discussion

The CC for PRR showed a significant swing in five of the participants. Although this was not the case for Participants C, E, F, and I, the CCs for MRAS and MNA, as well as PRR, exhibited similar systematic changes, except for the differences in characteristic frequencies. Accordingly, the lack of significance in the result may be due to the small sample size and insufficient variation or skewed distribution of the DD score (in the case of Participant J, the result was different in that the correlation with the DD score was rather weak also for MRAS and MNA, which may be due to the intake of antidepressants). It can therefore be concluded that the PRR varied in response to the DD score in many of the healthy participants.

The DD score indicates a depressed mood at the time of measurement, which can exhibit daily variations, rather than the severity of depression that usually does not change much in a short term. In addition, most of the observed DD scores were less than 10, indicating that the depressed mood was very mild and unlikely to affect cognitive functions. Consequently, changes in PRR at characteristic frequencies are likely to reflect short-term changes in depressed mood rather than changes in cognitive functions.

Although it has been pointed out that phase resetting may play an important role in information processing in the brain^13^ and previous studies reported that the duration and interval of phase resetting correlated with intelligence quotient^15^ and were different between autistic and normal children^16^, the relationship between phase resetting and depression has not been reported. In addition, although biomarkers for depression have been explored for many years, MRAS and MNA were not found to correlate with depressed mood. This is presumably because most previous studies have been based on inter-individual rather than intra-individual comparisons, despite the large individual differences, particularly in characteristic frequencies, and because most frequency-based analyses have focused on frequency bands, such as alpha and beta, whereas both positive and negative characteristic frequencies are often included in the same band.

Why and how does the PRR change in correlation with depressed mood? Phase resetting is unlikely to be a direct cause or consequence of depressed mood because PRR is minimum at approximately 10 Hz and maximum at approximately 15 Hz for all participants (Fig. 2b and Supplementary Fig. S1), where CC between PRR and the DD score was widely distributed from large negative to large positive values among participants, indicating that the absolute occurrence frequency of phase resetting is unrelated to depressed mood. Thus, some of the factors that cause phase resetting may be related to depressed mood.

A simple model is that as depressed mood increases, the EEG activity at positive characteristic frequencies becomes weaker and more unstable so that phase resetting can occur more easily and vice versa at negative characteristic frequencies. This model is consistent with the negative and positive CC for MNA at the positive and negative characteristic frequencies, respectively. It also explains the shift of the CC curve for MRAS to the right as follows: at a negative characteristic frequency, for example, as the depressed mood becomes lower and EEG activity is stronger, the phase angular speed at some higher frequencies is affected and reduced, which decreases the CC for MRAS and shifts the negative peak to the right. However, this model does not explain some cases in which the absolute value of the CC for PRR was large but was not large for MNA and also does not explain the systematic changes in the CCs.

Another possible model is the EEG frequency at which an extracortical input related to a depressed mood produces phase resetting shifts according to the depressed mood. This model can explain the CC curves for MNA and MRAS in the same way as the above model because phase resetting generally suppresses an increase in the amplitude of the EEG. Moreover, it explains the systematic changes in CCs to some extent because, in this model, an increase in PRR at a frequency is always accompanied by a decrease at another frequency.

Nevertheless, the latter model does not explain the geometric progression of peak frequencies. It should be noted that EEG activities at widely different frequencies are generally independent, and in fact, phase resetting rarely occurred simultaneously at one frequency and another (e.g., its doubled) frequency. Thus, further studies are needed to understand this phenomenon better. In addition, large individual differences in the characteristic frequencies are unexplained. Accordingly, unknown underlying mechanisms seem to mediate depressed mood and EEG activity.

In addition to the lack of details regarding the mechanisms underlying the observed effect, this study had several limitations. Firstly, it was a pilot study with a small number of participants. The participants were apparently healthy, and their medical history was not checked prior to enrolment. Therefore, the effects of depression were not investigated, and long-term variables such as medication history were not controlled. In addition, the sample size (number of measurements) was insufficient in the case of some of the participants, particularly in those with a very narrow or skewed distribution of DD scores.

Secondly, we used a portable EEG device with a lower sampling frequency (128 Hz) and fewer (14) electrodes than those usually used in the laboratory. Consequently, we did not investigate the gamma-band activity, which may provide objective information on major depressive disease status^17^, and EEG from other electrode positions, particularly those in the central region, such as Fz, Pz, and Oz, which may be more suitable for observing EEG activities related to depressed mood. However, the fact that the observation could even be performed with such a low-cost device is of great practical relevance.

Thirdly, experimental conditions were not completely controlled. The measurements were conducted by the participants at their homes and the electrodes were not strictly positioned—electrode placement may have fluctuated with each measurement. In addition, the environment, such as the time of day, location, and sound, was not necessarily constant. Measuring under more controlled conditions may have allowed us to better characterise the association between EEG activity and depressed mood. In fact, Participant H, from whom the clearest result was obtained, was a researcher and had previous experience of EEG measurement.

Lastly, this study does not immediately provide a practical biomarker for depression for the following reasons: First, the identification of characteristic frequencies is necessary to estimate depressed mood; however, this currently requires several different EEG measurements as well as the completion of questionnaires. Second, high levels of depressed mood are not necessarily caused by depression. It is desirable to capture EEG activities that reflect not only depressed mood at the time of the measurement but also the progression of depression. Third, PRR cannot be directly compared between people because of individual differences, and novel methods need to be developed to compensate for these differences. Fourth, characteristic frequencies or other properties may vary with age or with the progression of depression. If this is the case, additional methods will also be required to compensate for such changes over time.

To address these issues and develop a depression biomarker that can facilitate the early detection of depression, further studies involving a larger number of participants and including patients with depression, as well as long-term experiments are required. Nevertheless, PRR is currently the only candidate that can estimate changes in depressed mood in individuals who are not yet diagnosed as being depressed, which seems essential for early detection of depression, and appears more promising than other candidates that can only discriminate EEG between patients with depression and healthy controls. Our findings may also contribute to the elucidation of depression mechanisms and the improvement and development of treatments for depression.

### Supplementary Information

Supplementary Figures.

## Data Availability

The raw data necessary to reproduce the results presented in this manuscript is available at https://doi.org/10.6084/m9.figshare.21929871.
