# Supplementary material for: Brainwave activities reflecting depressed mood: a pilot study
Source: Sci Rep. 2023 Sep 4;13:14036. doi: 10.1038/s41598-023-40582-y (PMC10477265; doi:10.1038/s41598-023-40582-y)
Supplement: Supplementary file 1 — Supplementary Figures. [file 41598_2023_40582_MOESM1_ESM.pdf]

Brainwave activities reflecting depressed mood: A pilot study

Masahiko Morita, Ryusei Otsu, and Masahiro Kawasaki

Supplementary Figures

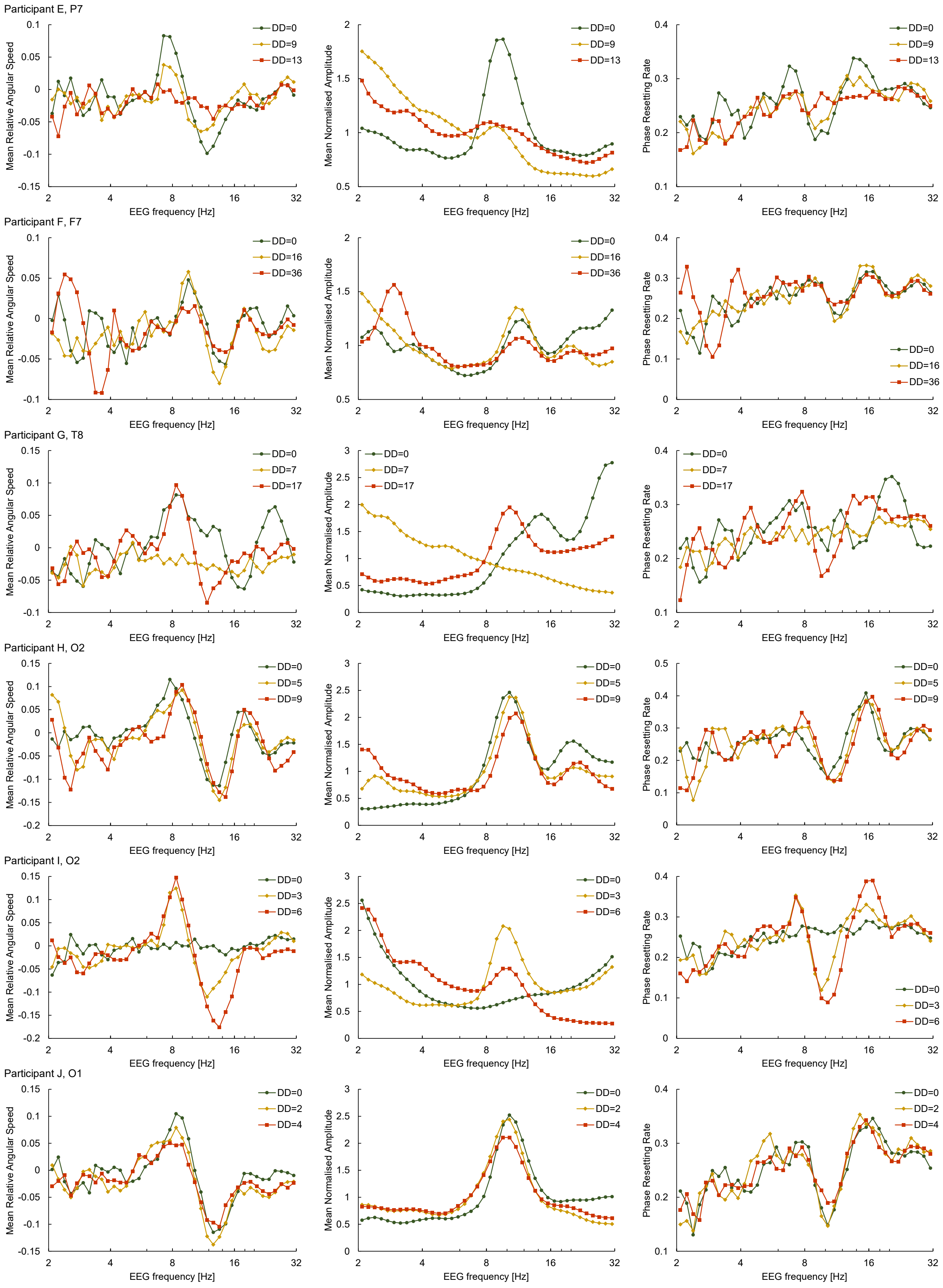

Figure S1: Mean relative angular speed, mean normalised amplitude, and phase resetting rate versus electroencephalogram (EEG) frequency for Participants E–J. See **Fig. 2b** for the description.

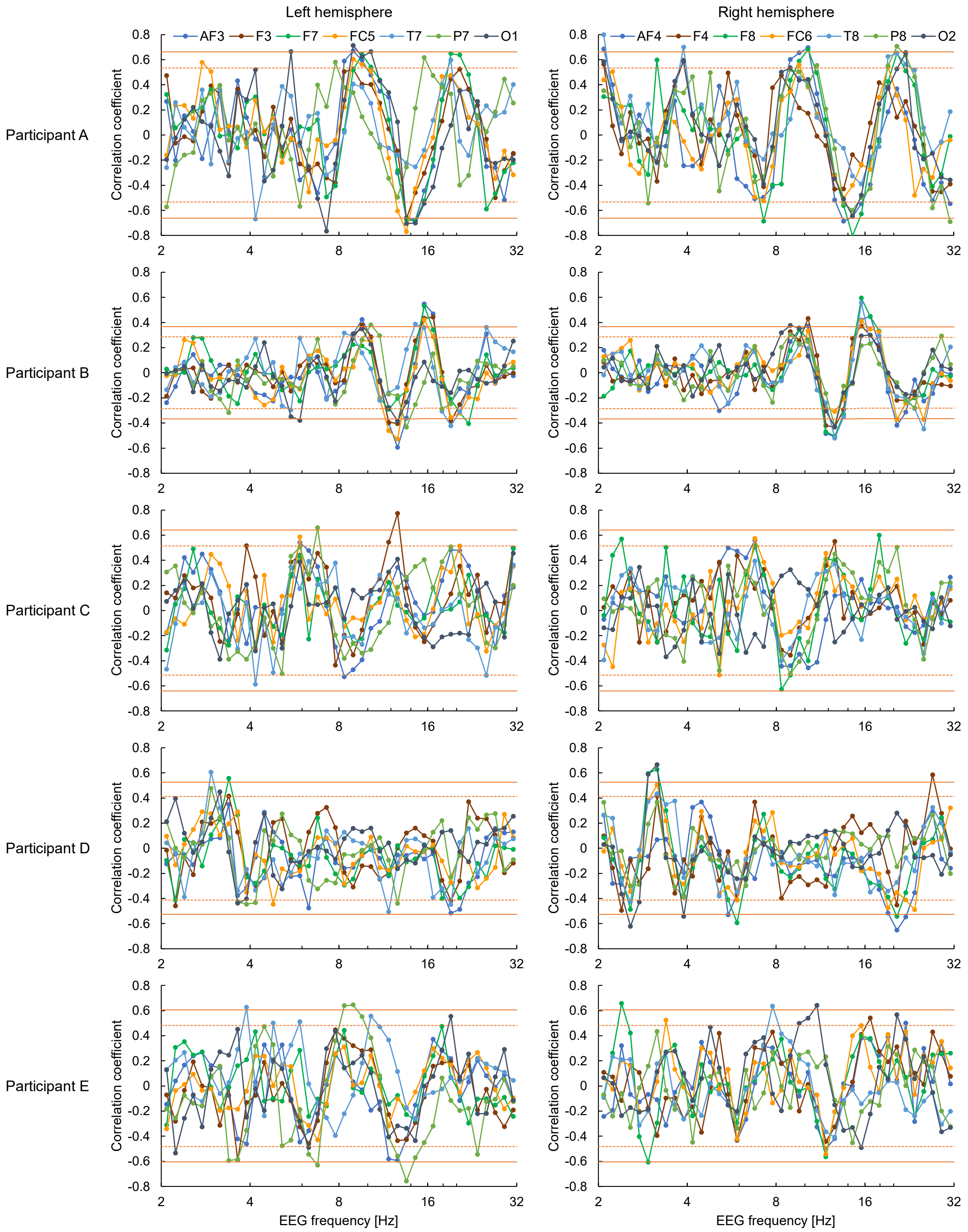

Figure S2: Correlation coefficients of phase resetting rate with Depression–Dejection score for all channels from Participants A–E. The orange solid and dotted lines are reference lines indicating *p*-values of 0.01 and 0.05, respectively.

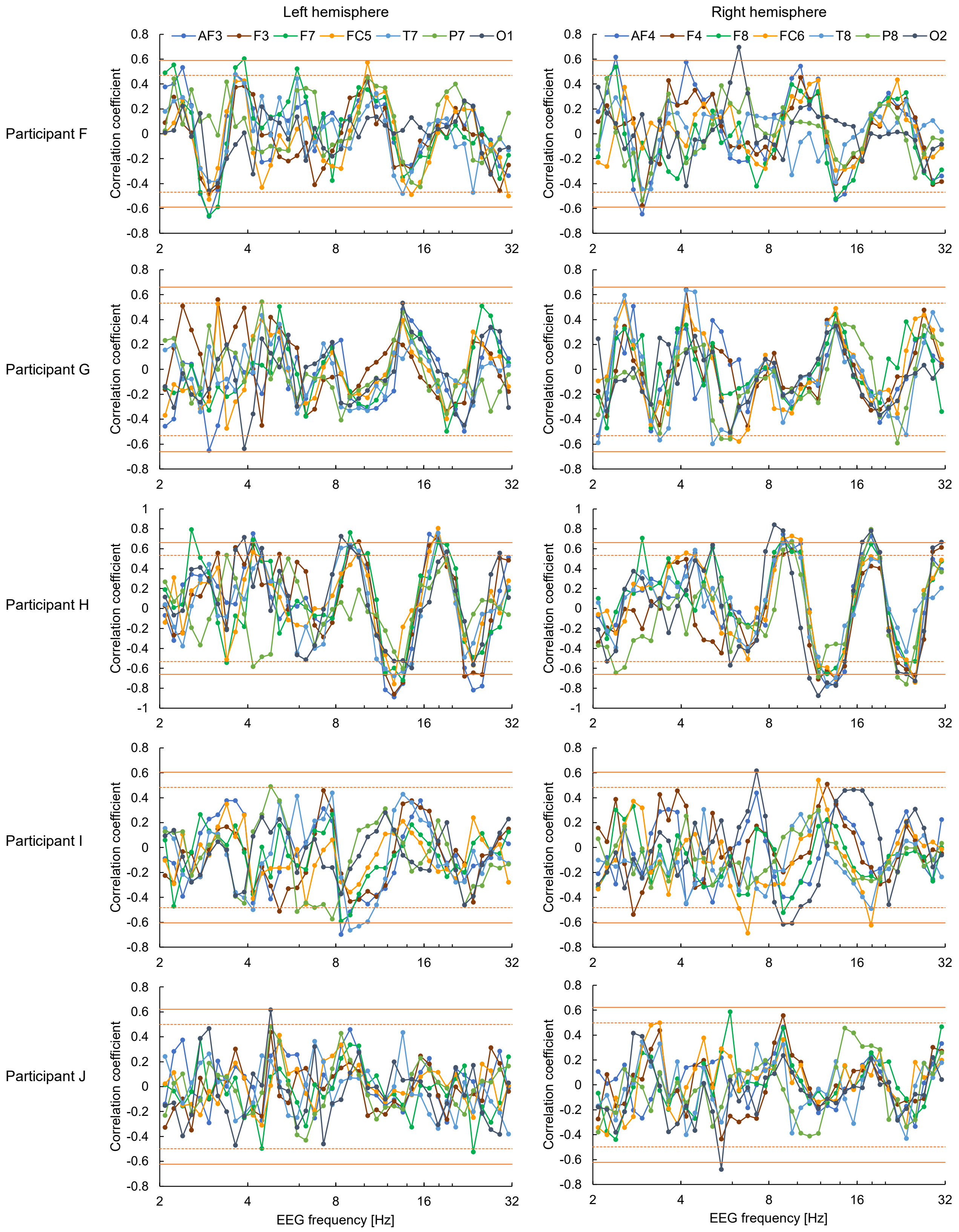

Figure S3: Correlation coefficients of phase resetting rate with Depression–Dejection score for all channels from Participants F–J.
